# Challenges to pooling models of crowding: Implications for visual mechanisms

**DOI:** 10.1167/19.7.15

**Published:** 2019-07-26

**Authors:** Ruth Rosenholtz, Dian Yu, Shaiyan Keshvari

**Affiliations:** rruth@mit.edu; dyumit17@gmail.com; shaiyan.o.keshvari@gmail.com; Computer Science and Artificial Intelligence Laboratory, Massachusetts Institute of Technology, Cambridge, MA, USA; Department of Brain & Cognitive Sciences, Massachusetts Institute of Technology, Cambridge, MA, USA; Computer Science and Artificial Intelligence Laboratory, Massachusetts Institute of Technology, Cambridge, MA, USA; Computer Science and Artificial Intelligence Laboratory, Massachusetts Institute of Technology, Cambridge, MA, USA; Department of Brain & Cognitive Sciences, Massachusetts Institute of Technology, Cambridge, MA, USA

**Keywords:** peripheral vision, crowding, pooling mechanism, high-dimensional pooling models

## Abstract

A set of phenomena known as *crowding* reveal peripheral vision's vulnerability in the face of clutter. Crowding is important both because of its ubiquity, making it relevant for many real-world tasks and stimuli, and because of the window it provides onto mechanisms of visual processing. Here we focus on models of the underlying mechanisms. This review centers on a popular class of models known as pooling models, as well as the phenomenology that appears to challenge a pooling account. Using a candidate high-dimensional pooling model, we gain intuitions about whether a pooling model suffices and reexamine the logic behind the pooling challenges. We show that pooling mechanisms can yield substitution phenomena and therefore predict better performance judging the properties of a set versus a particular item. Pooling models can also exhibit some similarity effects without requiring mechanisms that pool at multiple levels of processing, and without constraining pooling to a particular perceptual group. Moreover, we argue that other similarity effects may in part be due to noncrowding influences like cuing. Unlike low-dimensional straw-man pooling models, high-dimensional pooling preserves rich information about the stimulus, which may be sufficient to support high-level processing. To gain insights into the implications for pooling mechanisms, one needs a candidate high-dimensional pooling model and cannot rely on intuitions from low-dimensional models. Furthermore, to uncover the mechanisms of crowding, experiments need to separate encoding from decision effects. While future work must quantitatively examine all of the challenges to a high-dimensional pooling account, insights from a candidate model allow us to conclude that a high-dimensional pooling mechanism remains viable as a model of the loss of information leading to crowding.

## Introduction

### The puzzle of visual crowding

**Figure 1:**
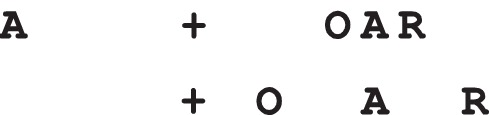
Visual crowding. (top) Fixate the + and try to identify the As on the left and right. (bottom) At a critical spacing, identification of the A improves.

In the fovea (i.e., the central rod-free area of the retina, approximately 1.7° diameter), recognition of visual forms is relatively robust and effortless. This is not the case for the 99% of the visual field outside the fovea. It is well known that the visual system has trouble recognizing peripheral objects in the presence of nearby flanking stimuli, a phenomenon known as *crowding* (Levi, 2008; Pelli & Tillman, 2008; Whitney & Levi, 2011). A classic demonstration can be seen in Figure 1. Fixating the upper cross, one can likely easily identify the isolated A on the left but not the one flanked by additional letters. An observer might see that there is an A in the string but not at its correct location—for example, to the right of the R. They might not see an A at all, or might see strange letterlike shapes made up of a mixture of parts from several letters (Lettvin, 1976). Move the neighboring letters—the *flankers*—farther from the target A, and at a certain *critical spacing* recognition is restored. The critical spacing is approximately 0.4 to 0.5 times the *eccentricity* (the distance from the center of fixation to the target) for a wide range of stimuli and tasks (Bouma, 1970; Pelli et al., 2009; Pelli, Palomares, & Majaj, 2004). Pelli and Tillman (2008) have dubbed this *Bouma's law*. This roughly linear dependence on eccentricity means that moving the display closer to or farther from the eyes has little effect on the critical spacing of crowding, over a wide range of viewing distances, which the reader can observe with Figure 1.

Crowding phenomena cannot be attributed to the peripheral loss of acuity (Bouma, 1970). Rather, they highlight peripheral vision's vulnerability to the presence of clutter. Even the earliest descriptions of crowding noted unique and interesting features distinct from loss of acuity. Korte (1923) described that under conditions of crowding, firm localization of detail becomes extremely difficult. Lettvin (1976) remarked, “It is not as if these things go out of focus—but rather it's as if somehow they lose the quality of ‘form'” (p. 10). A peripherally viewed word “only seems to have a ‘statistical' existence…[preserving] every property save that of the spatial order that would confer shape” (Lettvin, 1976, p. 14).

Crowding affects many real-world visual stimuli and tasks. It is not only relevant for recognition of arrays of items such as letters. Self-crowding can also occur, in which a single object can be sufficiently complex to be cluttered on its own, impairing recognition even without the presence of nominal flankers (Ehinger & Rosenholtz, 2016; Martelli, Majaj, & Pelli, 2005). Crowding has a far greater impact on perception than the peripheral loss of acuity or color vision, and it is the dominant difference between foveal and peripheral vision (Rosenholtz, 2016). It impacts visual search, object recognition, scene perception, perceptual grouping, shape perception, and reading (e.g., Pelli & Tillman, 2008; Pelli et al., 2007; Rosenholtz, Huang, & Ehinger, 2012; Rosenholtz, Huang, Raj, Balas, & Ilie, 2012). The information that survives crowding must suffice to guide eye movements and give us a coherent view of the visual world (Rolfs, Jonikaitis, Deubel, & Cavanagh, 2011). Its pervasive effects mean that we cannot hope to understand much of vision without understanding, controlling for, or otherwise accounting for the mechanisms of visual crowding.

**Figure 2:**
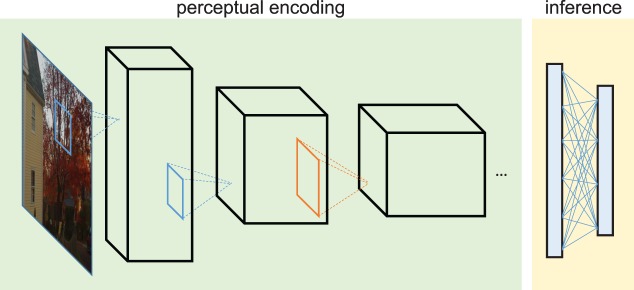
A candidate architecture for object-recognition processing. Each visual input proceeds through a series of encoding stages that gradually advance understanding of its contents. The encoding likely favors easy performance of ecologically relevant tasks at the expense of performance of other tasks (DiCarlo & Cox, 2007). Each stage may also lose information, perhaps because of limited resources. Finally, the organism makes inferences about the visual world. The observer may have more information for some decisions than others, making some tasks inherently easier. Object recognition could be difficult because of any of these stages of processing. The goal of understanding crowding is to uncover the mechanisms particular to crowding phenomena. Standard pooling models of crowding presume that crowding results from losses at a single stage of processing—e.g., as indicated in orange.

A challenge in understanding the mechanisms underlying crowding is distinguishing those mechanisms from the rest of object-recognition processing. In crowding experiments, we present a stimulus to the experimental subject and observe the outcome of the entire processing pipeline (Figure 2). A given condition could be difficult because of any of these stages of processing. We do not aim to elucidate the entire process for recognizing crowded objects (Tyler & Likova, 2007), nor for performing visual tasks more generally, but rather to model an important bottleneck in visual processing and thus understand what information survives and how that influences decision making and predicts the difficulty of visual tasks.

### A dominant theory of crowding: Pooling models

Crowding phenomenology—the jumbling, the loss of location information, and the seemingly statistical nature of the perceived stimulus—have pointed a number of researchers toward one particular class of crowding mechanisms. Crowding has been attributed to *excessive* or *faulty feature integration*, to *compulsory averaging*, or to *forced texture processing* (Balas, Nakano, & Rosenholtz, 2009; Lettvin, 1976; Levi, 2008; Parkes, Lund, Angelucci, Solomon, & Morgan, 2001; Pelli & Tillman, 2008), resulting from *pooling* over local regions (Balas et al., 2009; Parkes et al., 2001; Pelli et al., 2004). Pooling has typically been taken to mean averaging (Parkes et al., 2001) or otherwise computing summary statistics (Balas et al., 2009; Lettvin, 1976) of features within the local region. Despite differences in terminology, these descriptions appear to refer to similar theories: So-called *excessive* integration—over a region beyond the bounds of the target object—can be thought of as averaging or pooling over a sizable area of the visual field, and the operations involved in computing summary statistics are similar to mechanisms proposed to account for texture perception (for a review, see Rosenholtz, 2014). This class of crowding model is commonly known as a *pooling model*.

A fair assessment of the current state of the field is that pooling models dominate theories of crowding. These models are often not well specified, but we can infer a few critical attributes both from implemented pooling models and from research that claims to challenge a straightforward pooling account: First, pooling regions subtend sizable areas of the visual field and grow linearly with eccentricity (Bouma, 1970). Second, in straightforward versions of pooling models pooling occurs on a single processing level with pooling regions that are fixed in size, rather than changing with the stimulus or task. Although peripheral object recognition no doubt requires processing at multiple levels of a visual-processing pipeline, crowding models both from our own lab (e.g., Balas et al., 2009; Rosenholtz, Huang, & Ehinger, 2012) and from Freeman and Simoncelli (2011) explain crowding phenomena with fixed pooling at a single level (see also Pelli et al., 2004). Furthermore, arguments against a straightforward pooling account have explicitly criticized this assumption of fixed pooling regions at a single processing level (e.g., Kimchi & Pirkner, 2015; Louie, Bressler, & Whitney, 2007; Malania, Herzog, & Westheimer, 2007). Third, pooling regions overlap, and sparsely tile the visual field. In other words, neighboring pooling regions of a particular class—that is, that pool the same feature—do not exist at every possible spatial location. Rather, while neighboring regions overlap, their centers are separated by some distance (Balas et al., 2009; Freeman & Simoncelli, 2011). Pooling over sparse, sizable regions loses information, meaning one cannot generally reconstruct the visual input. Without sparseness and the resulting loss of information, pooling models would predict no crowding. Finally, we assume that after pooling, visual processing continues with whatever information remains.

If pooling occurs at a single level of processing, it is natural to ask at what level. Researchers have found evidence from adaptation studies that this level lies beyond V1 (He & Cavanagh, 1996; Liu, Jiang, Sun, & He, 2009; although see Nandy & Tjan, 2012), and have argued that pooling occurs shortly after early feature detection, in some sort of *feature integration* stage (Pelli et al., 2004; Pelli & Tillman, 2008). A number of implemented pooling models of crowding either explicitly or implicitly (through their choice of mechanisms) assume that pooling occurs after V1 (Balas et al., 2009; Freeman & Simoncelli, 2011; Parkes et al., 2001; van den Berg, Johnson, Anton, Schepers, & Cornelissen, 2012).

Crowding impairs many visual tasks, and yet peripheral vision supports a rich percept of the visual world. In order for a pooling model to be viable, it must predict both the limitations and the capabilities of visual perception. As a result, Rosenholtz (2014) has argued that we must make two additional assumptions. First, a pooling model must pool a large number of features, meaning the mechanism must involve a large number of populations of receptive fields (say, on the order of 1,000), with each population pooling a different feature. Second, Rosenholtz argues (and for the purposes of this article we assume) that the mechanism pools image features. In other words, it pools the outputs of filtering operations plus nonlinearities, as opposed to averaging the features of individuated *items*. An item-based model might, for example, extract the orientation of each bar in an array and average those orientations. Using object features can simplify modeling; for example, one can more easily construct an ideal observer for observations consisting of a discrete set of item features (Parkes et al., 2001; van den Berg et al., 2012) than for continuous outputs of image-processing operations. However, while one can certainly make interesting progress by studying object-based models (van den Berg et al., 2012), ultimately there are limits to the generalizability of such models (Keshvari & Rosenholtz, 2016). To distinguish the class of models that pool large numbers of image features from a pervasive *simple pooling model* (e.g., Greenwood, Bex, & Dakin, 2009, 2012; Levi & Carney, 2009; Parkes et al., 2001) that pools small numbers of features or item features, we call the former high-dimensional (HD) pooling model.

Work from Rosenholtz and colleagues (Balas et al., 2009; Ehinger & Rosenholtz, 2016; Keshvari & Rosenholtz, 2016; Rosenholtz, Huang, & Ehinger, 2012; Rosenholtz, Huang, Raj, et al., 2012; X. Zhang, Huang, Yigit-Elliot, & Rosenholtz, 2015), has developed and tested an HD pooling model that we call the Texture Tiling Model (TTM).^1^ The model consists of two stages. In the first stage, TTM implements a V1-like representation consisting of responses to oriented, multiscale feature detectors. In the second stage, the model computes a large set of second-order correlations from the responses of that the stage, taking the average over local pooling regions (TTM also computes more basic first-order summary statistics within each color band; Balas et al., 2009). These pooling regions grow linearly with eccentricity, in accord with Bouma's law, and overlap and tile the visual field. The information encoded in the second stage, where pooling happens, has been associated with the information encoded physiologically, post-V1 (e.g., Freeman, Ziemba, Heeger, Simoncelli, & Movshon, 2013; Yamins & DiCarlo, 2016). In addition, standard models of hierarchical visual processing (e.g., Fukushima, 1980; Riesenhuber & Poggio, 1999) often have as a second stage the computation of co-occurrence of combinations of features from the first stage; second-order correlations are merely co-occurrence computations pooled over significantly larger regions. The set of statistics we measure are those identified by Portilla and Simoncelli (2000), because that set has been successful at capturing the appearance of textures for human perception. Specifically, textures synthesized using this set of statistics are often difficult to discriminate from the original (Balas, 2006). Mounting evidence supports TTM as a good candidate HD pooling model for the peripheral encoding underlying crowding. We have shown that it predicts performance at a range of peripheral recognition tasks involving arrays of letters and other symbols (Balas et al., 2009; Keshvari & Rosenholtz, 2016; Rosenholtz, Huang, & Ehinger, 2012; Rosenholtz, Huang, Raj, et al., 2012). The same model predicts the difficulty getting the gist of a scene when fixating—that is, when forced to use extrafoveal vision—compared to when free-viewing that scene (Ehinger & Rosenholtz, 2016). With the same image statistics but a somewhat different arrangement of pooling regions, Freeman and Simoncelli (2011) have predicted the critical spacing of crowding. They have also shown that equating those local summary statistics creates synthetic metamer images that are difficult to distinguish one from another when viewed with the same fixation as used by the model (though see Wallis, Bethge, & Wichmann, 2016). While in all of these studies there has remained variance unexplained by the model, and thus room for improvement, these HD pooling models have so far proven quite powerful at capturing crowding and related visual phenomena.

### Challenges to a pooling account of crowding

In spite of the success of HD pooling models, however, questions remain. Behavioral researchers have made considerable progress understanding crowding in the last 1.5 decades. They have substantially expanded crowding phenomenology to a wider array of stimuli and tasks, moving well beyond arrays of letters and Gabors, to include stimuli with higher level grouping effects and tasks with complex naturalistic stimuli. As researchers have studied a wider range of stimuli and tasks, a complex pattern of results has emerged. A number of challenges have arisen to the relatively simple pooling model, and it has seemed that a single unifying explanation might not suffice. Researchers have called into question virtually every feature of pooling models highlighted in the foregoing, and instead proposed that more complex and often more dynamic models may be necessary. This review article centers on these challenges to pooling models.

Some behavioral results have seemed to favor a different type of mechanism entirely, for example the substitution mechanisms described in more detail in the next section (Strasburger, 2005; van den Berg et al., 2012). Other results have seemed to suggest that information is not lost, as it would be by a pooling mechanism, but rather rendered unavailable for object recognition (Chaney, Fischer, & Whitney, 2014; Yeh, He, & Cavanagh, 2012). Finally, other results have appeared to point to an attentional rather than a pooling mechanism (Intriligator & Cavanagh, 2001).

Other challenges have pointed to pooling operating at a different level of processing. For instance, Levi and Carney (2009) have suggested that pooling might follow segmentation into objects, perhaps also implying the pooling of object features rather than image features. Or perhaps multiple bottlenecks limit peripheral processing, rather than a single bottleneck. Researchers have suggested that crowding mechanisms might operate at multiple levels of processing rather than at a single feature-integration stage (Farzin, Rivera, & Whitney, 2009; Ikeda, Watanabe, & Cavanagh, 2013; Kimchi & Pirkner, 2015; Louie et al., 2007).

Finally, other challenges have suggested that pooling regions, rather than being fixed, vary with the stimulus and task (Banks & White, 1984; Bernard & Chung, 2011; Kimchi & Pirkner, 2015; Livne & Sagi, 2007; Manassi, Lonchampt, Clarke, & Herzog, 2016; Manassi, Sayim, & Herzog, 2012; Rosen & Pelli, 2015). In the most popular version of this suggestion, pooling occurs only within perceptual groups (Banks & White, 1984; Manassi et al., 2012).

We refer to the set of phenomena challenging a unified pooling-model account as the *model challenges*. We discuss each of these in more detail in the following sections. It is natural to think that this complex set of phenomena rules out a unifying pooling-model explanation. However, upon closer consideration, this may not be the case. We will argue that pooling models remain viable, in spite of numerous challenges.

## Challenge 1: The mechanism of crowding is not pooling but substitution

An observer faced with an array of items such as letters and asked to report the identity of a target may instead report one of its flankers. Such substitution phenomena are well known and well documented (Huckauf & Heller, 2002; Poder & Wagemans, 2007; Strasburger, 2005). These phenomena at first glance appear to challenge a simple pooling model. Why, if one encodes the average feature, would one report the features of the flanker rather than the target? Relatedly, other researchers have observed, “None of our participants ever spontaneously reported seeing [the mean]. This argues against … averaging and [in favor of] an inability to accurately localize features” (van den Berg, Roerdink, & Cornelissen, 2007, p. 10). While in subsequent work (van den Berg et al., 2012; van den Berg et al., 2010) these authors are firmly in favor of pooling models (which they refer to as *integration* models), later we revisit this observation in order to clarify intuitions about what pooling models predict.

Some researchers have further suggested that substitution *phenomena* might arise from a substitution *mechanism*. For example, the visual system might measure the features and possibly even the identities of both target and flankers, but either not encode their locations at all or encode them in a noisy way (Chung & Legge, 2009; Strasburger & Malania, 2013; van den Berg et al., 2012). The loss of location information would predict substitution errors, as the observer would accidentally report the incorrect item.

A substitution mechanism would immediately have consequences for set perception. If peripheral vision preserves the identities of the display items but not their locations, this would make reporting set properties such as the mean orientation easier than recognizing the features of a particular target item. This prediction agrees with behavioral results (Fischer & Whitney, 2011; Parkes et al., 2001). Fischer and Whitney (2011), for instance, showed subjects a peripheral array of faces and asked for both the facial expression of the central target and the mean expression of the set. They found that even though subjects had trouble reporting the target facial expression, that expression nonetheless contributed to judgments of the mean expression. They argue that these results are incompatible with a pooling mechanism, reasoning that if pooling loses information about that central target, the information cannot also be available to contribute to perception of the mean.

### An HD pooling model can predict substitution behavior and good set perception

We argue that these apparent challenges to a pooling model arise from misunderstanding HD pooling. It is difficult to reason about an HD pooling model, particularly one that measures image rather than object features. Researchers have instead attempted to gain intuitions from lower dimensional models. However, an HD pooling model will behave fundamentally differently from its low-dimensional brethren.

**Figure 3:**
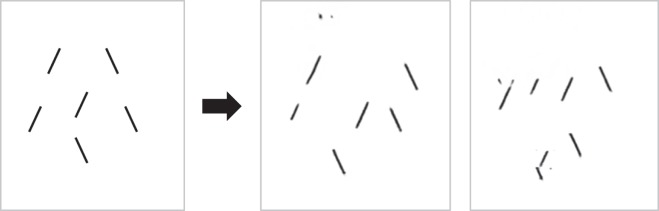
Original image (left) and two members of the equivalence class of our candidate high-dimensional pooling model, the Texture Tiling Model (right). The array is 3.6° in diameter, each item is 1° in length, and the fixation (not shown) is modeled at 10° to the right of the central target. A high-definition pooling model encodes a great deal of information about the stimulus yet also loses information, introducing uncertainty about the location and number of items.

**Figure 4:**
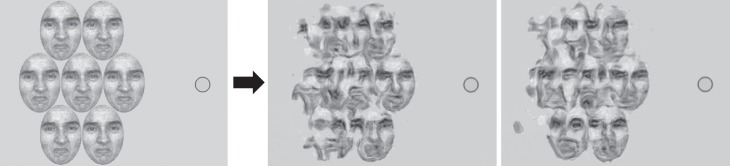
(left) Reprinted with permission from “Object-level visual information gets through the bottleneck of crowding,” by J. Fischer and D. Whitney, 2011, Journal of Neurophysiology, 106(3), p. 1390. Copyright 2011 by the American Physiological Society. The faces were adapted from face stimuli in Ekman's Pictures of Facial Affect (POFA) collection and are reprinted with permission from the Paul Ekman Group. (right) Two typical mongrels. Fixation and a 1° fovea indicated by the circle. The faces used in the emotional morph were drawn from Ekman's Pictures of Facial Affect collection and are reprinted in modified form with permission from the Paul Ekman Group.

To get intuitions about the information preserved and lost by an HD pooling model, we can generate members of the equivalence class of the model—that is, images that are confusable with the original, according to the model. Rosenholtz and colleagues have called these images *mongrels* (Balas et al., 2009). Consider the example in Figure 3. Loosely speaking, information that appears clear and unambiguous in these mongrels corresponds to information that survives HD pooling. Tasks that appear easy to perform with these visualizations are predicted by an HD pooling model to be easy tasks. For all of the examples in this article we generated at least 10 mongrels, and present one or two typical ones. For example, if the original task was to judge the orientation of a crowded peripheral target, we rank-ordered the mongrels according to our subjective assessment of the quality of the information available to perform that task and selected mongrels of median quality. To get a sense of the variability among the mongrels for a single input image, the full set of mongrels generated for Figures 3, 4, 7, and 16 are viewable in the supplementary material at https://dspace.mit.edu/handle/1721.1/121152. At that link can also be found the code for generating mongrels.

First and foremost, note that the encoding captures a great deal of information about the appearance of the stimulus. Sufficient information survives pooling to determine that the input consists of black lines against a white background. The model was not told anything about oriented black lines, but enough information survives pooling for later processes to get that gist.

Second, note the loss of location information. If the task were to discriminate the orientation of the central bar, observers might have trouble reporting that orientation rather than the orientation of one of the flankers. We can immediately see that a pooling model can predict substitution phenomena, at least qualitatively (Harrison & Bex, 2015; Keshvari & Rosenholtz, 2016).

To what extent swap errors occur in practice likely depends in part on the task, with different answers possible even with the same set of stimuli. Harrison and Bex (2015) found a low number of swap errors and suggested that those errors could be predicted by their population-code model. (Note that HD pooling models can also be considered population-code models.) Agaoglu and Chung (2016) found that with the same stimuli (concentric Cs), observers made considerably more swap errors—that is, reported the gap location from one C when asked about the gap location for a different C. That high number of swap errors likely arose from the complicated task, which required observers to report both ring orientations, with the order of the report varying from trial to trial and postcued. Harrison and Bex's experiments did not have this additional source of uncertainty. Additionally, for these stimuli it remains unclear how many swap errors TTM predicts. Agaoglu and Chung tested an early version of TTM, which used only a single pooling region and seeded synthesis with a blurry version of the original image. They showed that what they called the “texture synthesis model”^2^ rarely produces such substitution errors and concluded that TTM cannot predict the magnitude of substitution effects. However, it is worth noting that we originally used the blurry seed to *reduce* location uncertainty, as the single-pooling-region version of the model preserves *no absolute location information* (Balas et al., 2009). We no longer use this technique in the full version of TTM with multiple pooling regions. It is an open question whether TTM predicts more swap errors for these stimuli.

The third thing we can note from Figure 3 is that, for this simple display, pooling preserves enough information to determine the distribution of orientations fairly accurately. An observer asked to report the orientation of the central target would have no reason to report it as vertical, as the representation makes clear that the stimulus contains no vertical lines. If this were the information available in the periphery, observers should be good at reporting all sorts of set properties. Once pooling loses location information, set perception becomes inherently easier than reporting a particular item. The visual system has information about the set, but information about a given item becomes inaccessible in the sense of it being difficult to determine which item is the desired target.

This general logic generalizes to other kinds of set perception, such as mean facial expression (Figure 4). HD pooling has no difficulty predicting that an incorrectly identified target can contribute to the perceived mean. For such complex stimuli as those of Fischer and Whitney (2011), the more pressing question is whether our particular HD pooling model preserves enough information to predict judgments of mean facial expression at human accuracy levels. These mongrels suggest that HD pooling “render[s] further object processing” difficult, but not “impossible” (Fischer & Whitney, 2011, p. 1397). Clearly, set-perception performance—according to an HD pooling model—depends upon the complexity of the individual items and of the display.

Substitution phenomena do not eliminate pooling models. One might ask why, then, studies ever find averaging effects (e.g., Greenwood et al., 2009; Parkes et al., 2001). There are two reasons. First, when target and flanker features are sufficiently similar, representation of those features can become poorer. (See, for example, representation of the similar orientations in Figure 7.) The orientation of the central target appears to be a mix of that of the target and those of the flankers. Under such conditions, one might imagine that observers would report something like the mean feature, and they do (Greenwood et al., 2009).

**Figure 5:**
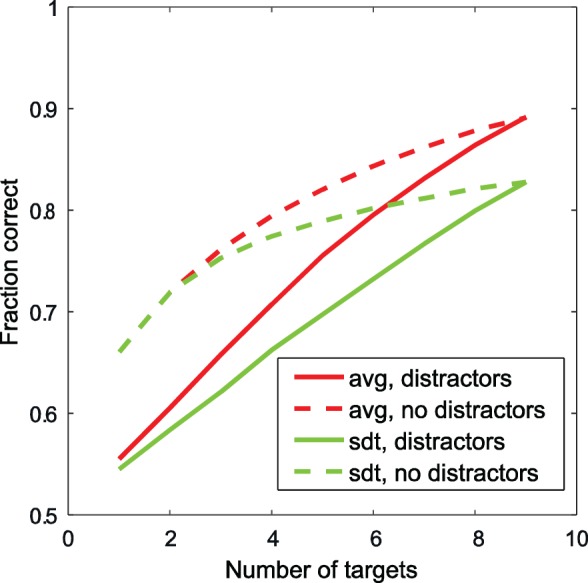
Performance of compulsory averaging and signal-detection-theory models with the same internal noise. Solid curves show predicted performance for the Parkes, Lund, Angelucci, Solomon, and Morgan (2001) condition in which stimuli contained both tilted targets and nontilted distractors. Dashed curves show predicted performance for the Parkes et al. condition in which stimuli contained only tilted targets. In both conditions the target tilt was 12.5° and the internal noise was Gaussian distributed with a standard deviation of 30°. Performance is always the same or better for the averaging model (red curves) than for the signal-detection-theory model (green curves). Rather than compulsory, averaging may be a good strategy for these particular conditions.

Second, observers may base their decision upon the average because doing so is a good strategy for a given task. In their seminal article, Parkes et al. (2001) showed observers one or more target Gabors, tilted clockwise or counterclockwise, and observers had to report the direction. All targets on a given trial had the same tilt. In one condition, *n* targets were present with no distractors. In the other condition, nine items appeared: *n* targets and 9 − *n* distractor Gabors with zero tilt. The researchers found that they could better fit the results with a model in which observers based their decision on the mean of the noisy orientation observations than with a signal-detection-theory (SDT) model in which observers retained observations for every Gabor. One might be tempted to conclude that an observer only has access to the mean (compulsory averaging). However, it turns out that responding based upon the mean orientation yields better results than the supposed SDT strategy described by Morgan and Solomon (2005).^3^ Their SDT strategy chooses the tilt direction by finding the maximum observation *M* (the most clockwise observation) and the minimum observation *m*. The model chooses the clockwise response if |*M*| > |*m*|, counterclockwise if not. This strategy is not ideal for a threshold experiment in which multiple targets deviate by the same amount (Ma & Huang, 2009). Intuitively, as the number of targets increases, the SDT model cannot make use of the additional information to estimate the tilt. On the other hand, the averaging model, which chooses clockwise if the average of the noisy observations is greater than 0, does make use of information from additional targets. Figure 5 shows for both models the predicted fraction correct as a function of the number of targets, given the same internal noise. The pattern of results is the same regardless of the internal noise (excluding extremes in which predicted performance is at ceiling or at floor for both models). An observer following the SDT strategy never performs better than one making their decision based on the average, for the Parkes et al. tasks. An optimal model, with access to the full but noisy distribution of orientations, would behave as if it had access only to the mean.

Much has been made of the apparent dichotomy between substitution and averaging behavior (Ester, Klee, & Awh, 2014; Freeman, Chakravarthi, & Pelli, 2012; Greenwood et al., 2009). However, pooling models can produce substitution phenomena, and models capable of producing substitution phenomena, in turn, should sometimes produce averaging behavior.

### An HD pooling model does not behave like a slots model

One way of implementing a substitution model would be to have *n* slots, one for each item in the stimulus and its features, akin to slots models of short-term memory (Zhang & Luck, 2008). Entire items might swap between slots, leading to classic substitution effects, or features might swap between slots, leading to more complex sorts of confusions.

The previous subsection discussed the fact that a pooling model can produce substitutionlike confusions. However, it should also be clear that a pooling model does not behave like a slots model. As we can see in Figure 3, pooling does not simply make items or their features swap positions with each other, but rather makes features and their locations ambiguous and confusable. Pooling can even produce ambiguity about the number of items present. Put another way, an HD pooling model operates on image features, not a list of items and their features; nor is the information available at the output of a pooling model simply a list of items and their features.

**Figure 6:**
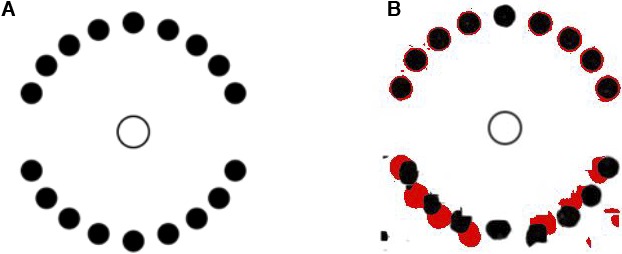
(A) Original image based on stimuli from Intriligator and Cavanagh (2001). (B) Two typical mongrels overlaid, with red indicating regions that were black in one mongrel but not the other. Fixation and a 1° fovea indicated by the circle.

Intriligator and Cavanagh (2001) attempted to distinguish between a pooling and an attentional-selection mechanism for crowding. We discuss their work as part of the substitution-related challenges because they presume that a pooling model acts like a slots-based substitution model. They cued one of a number of identical disks (Figure 6A), then instructed observers to move the focus of their attention from item to item in a prescribed way (“left-right-right-left-right…”). Then they asked observers to identify the item indicated by the cue plus series of instructions. They varied item spacing until observers reached threshold performance levels. The resulting critical spacing was similar to that of crowding. Thus, they suggested that crowding arises from an inability to selectively attend to the target. Of interest here is their claim that “mixing of adjacent features … cannot contribute to the critical spacing in our [study] where target features and identity are irrelevant” (p. 208). Their reasoning seems to presume that vision has some number of slots, each containing the features of one item; feature pooling mixes up the contents of the slots—irrelevant in this case, since the slots contain identical items—but not the slots themselves. As a result, they reason, their results cannot be due to pooling.

**Figure 7:**
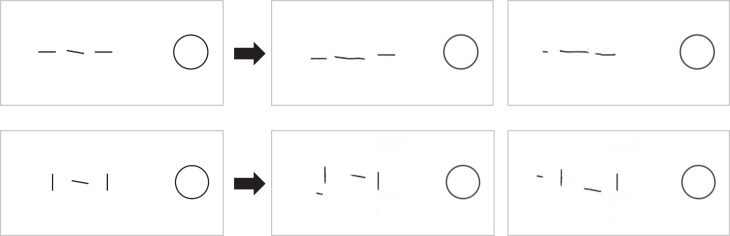
Orientation similarity effect. Determining the tilt of the central target is easier when flankers have dissimilar orientation (bottom) versus similar (top). In each row, the two images on the right show two mongrels, visualizations of the information available according to our high-definition pooling model. The target and its orientation are clearer in the two mongrels for the dissimilar condition; high-definition pooling better encodes the target in that condition. High-definition pooling predicts this orientation similarity effect without pooling at an orientation-processing stage.

In Figure 6B we have overlaid a pair of mongrels for Figure 6A, with red indicating regions that were black in one mongrel but not the other. The difference between the two mongrels demonstrates the position uncertainty inherent to the model. One might imagine that this degree of uncertainty would make attentionally tracking the target difficult. Because an HD pooling model does predict negative effects of crowding even when the display items are identical, one cannot rule it out as an explanation for the results of Intriligator and Cavanagh (2001).

## Challenge 2: Crowding arises from multilevel pooling

Straightforward versions of pooling models presume that crowding arises from pooling at a single critical level of processing. Some recent theories have suggested instead that crowding might involve pooling at multiple levels of processing. In addition to pooling soon after early feature detection, there might exist crowding mechanisms at the part-processing level or shape-processing level (Kimchi & Pirkner, 2015), the face-processing level (Farzin et al., 2009; Louie et al., 2007), and the point-light-walker-processing level (Ikeda et al., 2013). This is an attractive and rather intuitive idea, at first glance, because it parallels standard models of object recognition (e.g., Fukushima, 1980; Riesenhuber & Poggio, 1999). Standard hierarchical models of vision do involve multiple levels of processing, with some sort of pooling or integration at each level. However, while object-recognition models do usually alternate between filtering and pooling stages, the pooling typically occurs over an area not much larger than the size of the filter (Krizhevsky, Sutskever, & Hinton, 2012; Riesenhuber & Poggio, 1999), and thus might not cause crowding per se.

There is an immediate reason to be concerned about the suggestion that models of crowding need to incorporate multiple levels of pooling: Our candidate HD pooling model already loses a lot of information through pooling at a single level, and yet quantitatively predicts a number of phenomena. Our modeling thus far has not suggested the need to pool at additional levels, in spite of testing a relatively large variety of tasks and stimuli. If one were to pool at additional levels of processing, additional information would be lost, perhaps reducing the predictive power of the model. (Note that this particular criticism would not apply to the hierarchical model of Chaney et al., 2014. Rather than losing information at each level of processing, that model predominantly loses information at the decision stage, through a mechanism that can access only a sparse sampling of receptive fields in order to make a decision.) Here we reassess the multilevel-pooling claims both on a theoretical level and using intuitions from our HD pooling model.

Many claims of multilevel pooling involve similarity effects, in which it is easier to identify a target flanked by dissimilar items than one flanked by similar items. Such effects are prevalent in the crowding literature. Identifying a target letter is easier when it pops out from the flanker letters due to a difference in color (Scolari, Kohnen, Barton, & Awh, 2007), and similar effects have been found for large target–flanker differences in orientation (Andriessen & Bouma, 1976), contrast polarity, shape, and binocular disparity, but not eye of origin (Kooi, Toet, Tripathy, & Levi, 1994).

One theory about why these similarity effects occur is that pooling operates only within a feature band. (Alternatively, although the distinction is not critical for the present discussion, *inhibition* might occur only within a band; Andriessen & Bouma, 1976; Kooi et al., 1994; Levi, Hariharan, & Klein, 2002). According to this theory, if the stimulus has similar flankers, pooling mixes them in with the target, leading to crowding. Dissimilar flankers do not mix with the target, leading to a release from crowding. If this theory is correct, then a seductive corollary would seem to be that one can figure out where in visual processing it is that pooling (i.e., crowding) occurs by looking at what kind of similarity effects one finds. If one finds a shape similarity effect, then pooling must be at the shape-processing stage. This suggestion that one might uncover brain mechanisms through simple psychophysical experiments, coupled with standard models of hierarchical processing, makes multilevel crowding doubly attractive as a theory.

There are problems, however, with interpreting similarity effects in terms of the level of pooling. First, the theory presumes that recognition operates by having a receptive field tuned to the target object, reminiscent of grandmother cells, and that crowding arises because flankers lie within that receptive field, disrupting identification. But particularly at higher levels of processing, the encoding is likely more distributed. In a distributed encoding scheme, target identification arises through combining information from multiple feature detectors rather than from the response of a single band sensitive only to the target. Even in low-level vision, information from multiple receptive fields in V1, tuned to different orientations, *combines* to identify the underlying orientation.

Second, before using a similarity effect to reason about the level at which pooling occurs, one needs to confirm the level of (dis)similarity of target and flankers. Confirming that it is at a high rather than a low level—or, equivalently, controlling low-level similarity while varying high-level similarity—is notoriously difficult. Higher level classifications typically derive at least in part because of shared lower level features.

Both of these theoretical arguments suggest that there may be a mismatch between the apparent level of similarity between target and distractors and the pooling level that produces the effect. In fact, we see evidence of just that. Let us look at some mongrels to get intuitions about what an HD pooling model predicts. Our candidate HD pooling model largely pools pair-wise combinations of responses of V1-like orientation detectors. (Notable exceptions are its computation of marginal statistics of luminance and color.) One might think of it as pooling at the junction-processing level. Figure 7 shows a similar (top) and a dissimilar orientation (bottom) condition. The slightly tilted target line in the center is the same in both cases. On the right are two mongrels for each condition. What one should look for is how well these mongrels preserve the tilted target, and in particular how well one could judge its orientation. One can easily observe the more faithful representation of the target in the condition with dissimilar flankers. Our HD pooling model can at least qualitatively predict an orientation similarity effect (and in some cases quantitatively; see Keshvari & Rosenholtz, 2016), and yet it has no pooling at the orientation-processing stage. There is a mismatch between level of similarity and level of pooling.

**Figure 8:**
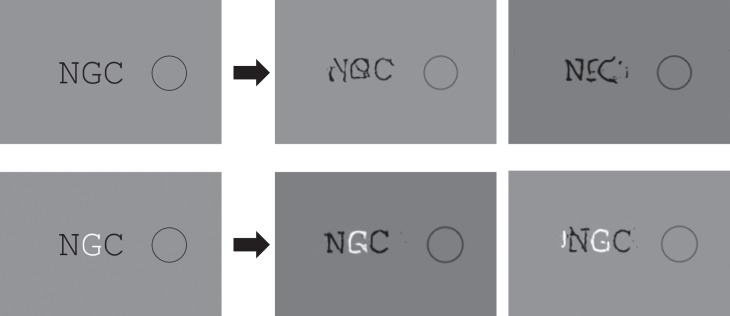
Sign-of-contrast similarity effect. Identifying the central letter is easier when flankers have an opposite sign of contrast (bottom). In each row, the two images on the right show two mongrels, visualizations of the information available according to our high-definition pooling model. The target's identity is clearer in the two mongrels for the dissimilar condition. High-definition pooling better encodes the target in that condition. High-definition pooling predicts a sign-of-contrast similarity effect without pooling at a sign-of-contrast processing stage.

For another example, consider the similarity effect in Figure 8, based on sign of contrast. It is easier to recognize the target (G) when it has a different sign of contrast than the flankers (Kooi et al., 1994). Looking at the mongrels, the G shape is quite well preserved in the dissimilar condition. Our HD pooling model predicts, at least qualitatively, a sign-of-contrast similarity effect, and yet it does not pool at a sign-of-contrast stage. There is a mismatch between the level of similarity and the level of pooling that produces the observed similarity effect.

**Figure 9:**
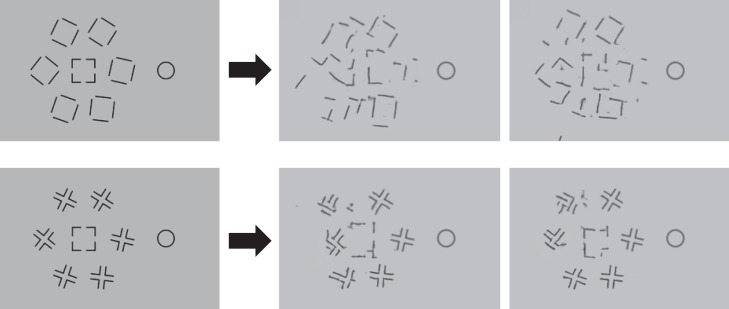
Kimchi and Pirkner (2015) found crowding both when a central square formed of L junctions was flanked by squares formed without L junctions (same shape, different parts, top left) and when it was flanked by L junctions that did not form a square (same parts, different shape, bottom left). However, this does not imply that crowding must happen at both the part and shape levels of processing. Mongrels on the right of the arrows show signs of crowding for both conditions. Crowding seems worse for the same-shape flankers, in agreement with experimental results for the modeled eccentricity of 5°. Original stimuli recreated based on stimuli from “Multiple level crowding: Crowding at the object parts level and at the object configural level,” by R. Kimchi & Y. Pirkner, 2015, Perception, 44(11), p. 1286, with permission from the author, R. Kimchi.

Finally, consider the shape similarity effect from Kimchi and Pirkner (2015). They flanked a target square composed of L junctions with a variety of flankers. Figure 9 gives two key examples. In the first condition, flankers have the same overall shape as the target, but consist of nominally different parts: straight lines instead of L junctions. In the second condition, flankers consist of the same L parts as the target, but those parts form different shapes. The observer indicated the orientation of the target—that is, whether it appeared as a diamond or a square. At the eccentricity shown, it is easier to recognize the square target when it is flanked by dissimilar shapes than by similar shapes. Again, the mongrels indicate that our HD pooling model better encodes the target in the dissimilar condition than in the similar condition; in other words, the HD pooling model predicts the shape similarity effect without any pooling at a shape-processing stage. Again, there is a mismatch between level of similarity and level of pooling.

**Figure 10:**
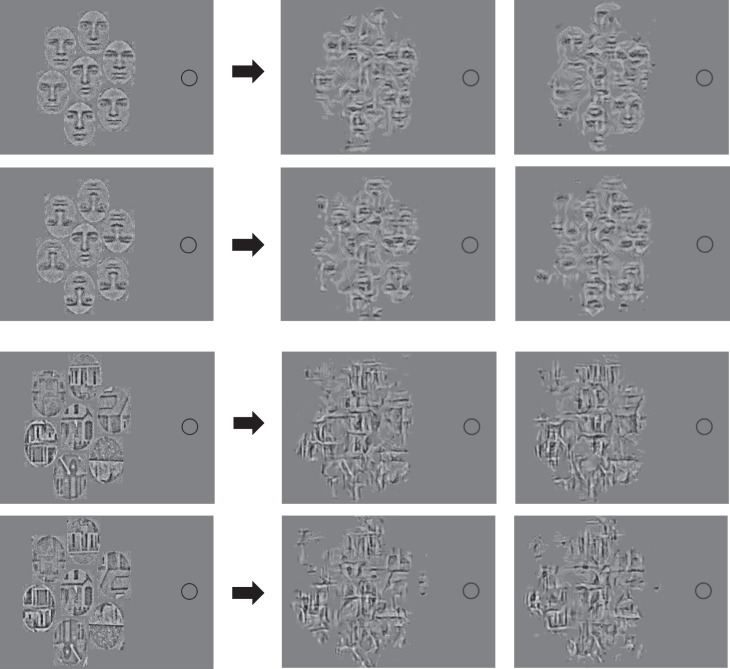
Original stimuli (left of the arrows) and their mongrels (right of the arrows) from Louie, Bressler, and Whitney (2007). The first two rows show target face surrounded by upright (first row) versus inverted (second row) flankers. The last two rows show a target house surrounded by upright (third row) versus inverted (fourth row) flankers. Original stimuli adapted from “Holistic crowding: Selective interference between configural representations of faces in crowded scenes,” by E. G. Louie, D. W. Bressler, and D. Whitney, 2007, Journal of Vision, 7(2):24, p. 3–4. Copyright 2007 by E. G. Louie, D. W. Bressler, and D. Whitney. Reprinted with permission.

Several articles from Whitney and colleagues have made a somewhat more complicated argument for crowding at a holistic face-processing level (Louie, Bressler, & Whitney, 2007; Farzin, Rivera, & Whitney, 2009). Louie et al. (2007) asked observers whether a target face appeared on the left or right side of the display, or not at all (Figure 10 shows a single side of the display). They found greater crowding when upright faces flanked the target than when inverted faces did. On the other hand, they found no effect of upright versus inverted flankers when the task was instead to detect a target house among house flankers (Figure 10). They argue that the difference between faces and houses may derive from holistic processing of faces versus part-based processing of houses. These results by themselves could arise from a relatively low-level similarity effect. Faces may show an effect of upright versus inverted flankers and houses not show such an asymmetry simply because there is less difference between an upright and an inverted house compared to an upright and an inverted face. Cropping the house stimuli further increases the similarity between upright and inverted houses, by eliminating distinguishing roof features. On the other hand, it does not seem obvious that TTM per se can predict the similarity effect. From the example mongrels in Figure 10, it appears difficult to identify the face or house in either condition, whereas in the original study observers had a *d*′ in the range of 2.5 to 3 for the upright face targets and around 3.5 for upright house targets. More quantitative study is needed, but we may find that TTM lacks necessary features to predict this relatively good performance. However the additional features that may be required are not obviously at the holistic face-processing level.

Louie et al. (2007), however, additionally argue for involvement of holistic face processing based on the results of repeating the two face conditions, but with the entire display inverted. The target faces then appeared upside down, and this manipulation eliminated the asymmetry between upright and inverted flankers. The researchers argue that flipping the stimuli should not change low-level similarity, concluding that their results arise from holistic face processing and that crowding can occur at the face-processing stage.

We agree that inverting the entire display should have minimal effect on low-level similarity but dispute that the results of Louie et al. (2007) necessitate that crowding operates at the holistic face-processing level. First, it is notable that Sun and Balas (2015) did not replicate the effect of inverting the entire display. They asked observers to categorize the gender of a target face and found crowding for a target flanked by facelike stimuli like line drawings and U.S. electrical sockets. Unlike Louie et al., Sun and Balas did find that the upright/inverted flanker asymmetry reversed when they inverted the target face, consistent with a low-level similarity effect.

Second, it is arguable that Louie et al. (2007) found no difference between upright and inverted flankers in their inverted face conditions because performance was near floor. Along these lines, Kalpadakis-Smith, Goffaux, and Greenwood (2018) systematically investigated the influence of task difficulty on face crowding. They asked observers to identify the horizontal separation between the eyes and found a similarity effect for both an upright and an inverted target face when the task was easy (large differences in interocular distance). However, when the task was difficult (small differences in interocular distance), they found no similarity effect for either upright or inverted targets. Whitney and colleagues have argued, however, that the lack of a holistic processing pattern of results may arise from using a nonholistic face task (Manassi & Whitney, 2018). However, Kalpadakis-Smith et al. showed that discriminating small differences in the horizontal separation between the eyes of a single face was indeed easier when the face was upright compared to inverted, following the pattern of a holistic task. Nevertheless, they found no difference between upright and inverted flankers when observers performed this task on a crowded face in their periphery.

Holistic processing may well be involved at a later stage; it operates on the information that survives crowding to produce better performance identifying an upright face than an inverted face. The lack of holistic processing for the inverted face conditions of Louie et al. (2007) likely led to the near-floor performance for inverted targets. However, we would not call this a crowding mechanism per se, as performance is better for upright faces even in the fovea. (Interestingly, visual search for a cube among differently lit cubes also has an asymmetry that does not persist when the entire display is inverted. In that case, we have similarly argued for a later loss of information due to estimating 3-D shape while discounting illumination; X. Zhang, et al. 2015. Again, we would not call this additional loss of information crowding, and in fact we saw evidence of this loss even in fixating individual, uncrowded cubes.)

**Figure 11:**
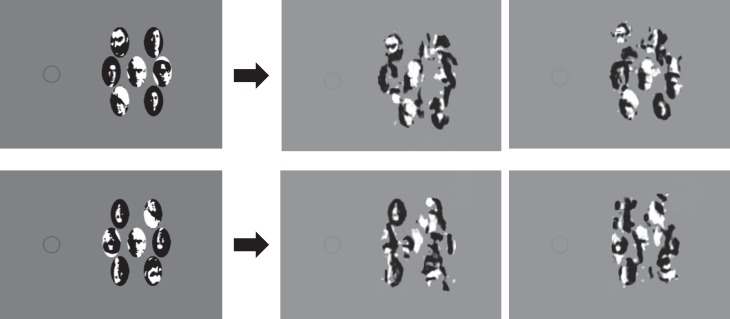
Original stimuli (left of the arrows) and their mongrels (right) from Farzin, Rivera, and Whitney (2009). Target faces are hard to identify when they are crowded by upright face flankers (top row) compared to inverted flankers (bottom row). Our model, without implementing a holistic face-processing mechanism, preserves some information for face tasks, as suggested by the mongrels. Arguably, it is also easier to guess the target face identity from the mongrels in the bottom row compared to the top, but further work with a larger set of stimuli would be necessary to quantify this. Original stimuli reprinted with permission from “Holistic crowding of Mooney faces,” by F. Farzin, S. M. Rivera, and D. Whitney, 2009, Journal of Vision, 9(6):18, p. 10. Copyright 2009 by F. Farzin, S. M. Rivera, and D. Whitney.

Farzin et al. (2009) have also argued for holistic face crowding, using somewhat different logic. They asked observers to perform a number of tasks with Mooney faces (e.g., judging the orientation or gender of the target) and found classic crowding effects (Figure 11). They also found a similarity effect: greater crowding of an upright Mooney face by upright flankers than by inverted ones. They did not test for an inversion effect, leaving open the possibility of a low-level similarity effect and low-level mechanisms. Rather, they argue against low-level crowding on the basis that Mooney-face tasks require holistic processing (Kanwisher, Tong, & Nakayama, 1998). Certainly, as illustrated in Figure 11, it is not obvious that TTM predicts the similarity effect. However, we argue that holistic face crowding does not logically follow. Processing Mooney faces requires processing at multiple levels prior to the supposed holistic processing. If recognition of Mooney faces fails under conditions of crowding, something must have gone wrong with one of those processing stages, but the fault does not obviously lie with the holistic processing stage. For an extreme example, just to make the point: If you closed your eyes and failed to identify the gender of a Mooney face, you would not blame the holistic processing stage.

In summary, one should be careful not to confuse phenomena with mechanisms. Just because crowding occurs when, for example, flankers have similar parts to the target, that does not mean that crowding occurs at the parts level of processing. Dakin, Cass, Greenwood, and Bex (2010) similarly argue that the seemingly object-level crowding effects they found may have a low-level explanation. One cannot easily reason from similarity effects to the stage at which pooling occurs. Nor can we rule out, at this time, the possibility that crowding is due to a single level of pooling.

## Challenge 3: Flexible pooling regions vary with the stimulus

**Figure 12:**
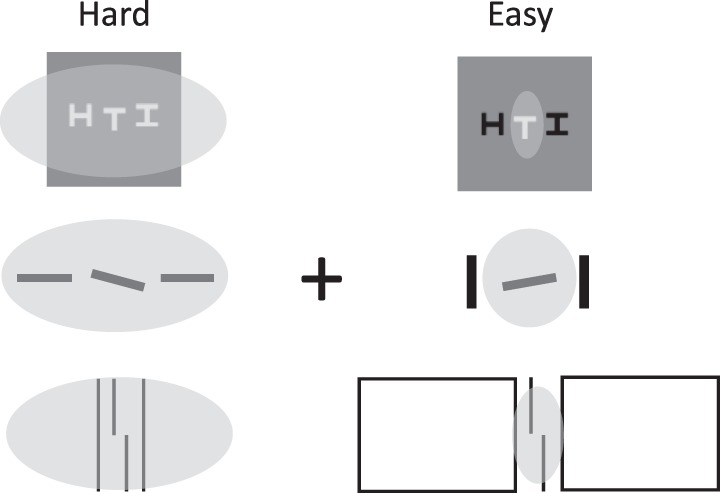
The flexible-pooling-region hypothesis. According to this hypothesis, when the target and flankers group together, they are pooled together (gray ellipses), resulting in crowding (left column). But when target and flankers are not grouped, they are pooled separately, resulting in less crowding (right column).

A third challenge to the pooling account of crowding suggests that pooling regions, rather than being static, might vary with the stimulus (Manassi et al., 2016; Manassi et al., 2012; Sayim, Westheimer, & Herzog, 2010). This challenge is based on an alternative theory of what causes similarity effects. According to this theory, the visual system pools only within a perceptual group. When the target and flankers group together (Figure 12, left column), the visual system pools over both target and flankers, leading to crowding and poor performance identifying the target. When, on the other hand, the target segments from the flankers (Figure 12, right column), the visual system pools over the target alone and flankers alone, leading to a reduction of crowding. As in the previous section, this theory presumes that relief from crowding arises from mechanisms pooling over the target and not the flankers. However, unlike the previous theory, the relief comes from dynamic adjustment of the region over which pooling occurs, rather than from narrow feature bands.

Once again, there is something attractive about the idea that pooling regions might adapt to the stimulus. What are grouping processes for, after all, if the visual system does not use them to intelligently process the stimulus?

**Figure 13:**
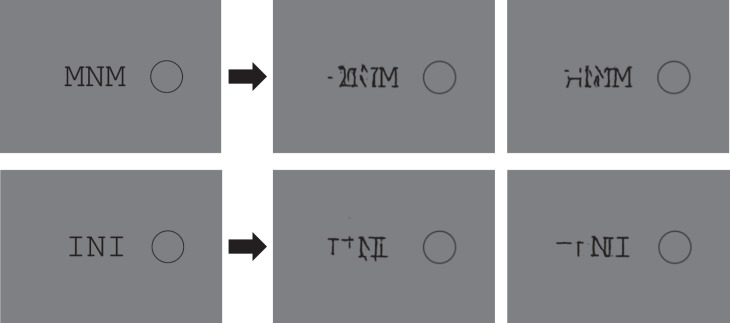
Similarity effect for letter shape. Identifying the central letter N is easier when it is flanked by dissimilar flankers (I) compared to similar flankers (M). High-definition pooling better encodes the target in the dissimilar condition, predicting the letter similarity effect without requiring a flexible pooling mechanism that adjusts to pool over only the target.

As with our discussion of multilevel crowding mechanisms, one must ask whether an HD pooling model can predict the effects without requiring a more complex mechanism (flexible pooling). We have already demonstrated that it can in some conditions, such as the sign-of-contrast example in Figure 8. Similarly, Keshvari and Rosenholtz (2016) have shown that an HD pooling model can predict letter similarity effects without the need for flexible pooling. As a demonstration, consider the stimuli in Figure 13. The target is the letter N in both cases, but the flankers are similar in the top condition and dissimilar in the bottom. The target N is better represented in the dissimilar case, suggesting that it will be easier to recognize, even with a fixed pooling mechanism.

**Figure 14:**
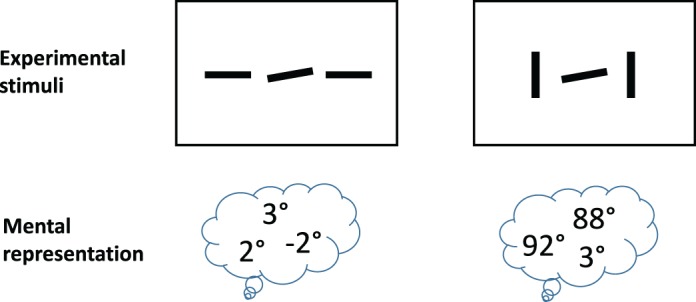
Under conditions of crowding, a pooling model might preserve information necessary to obtain noisy observations of the item features but not preserve enough location information to tell which observation goes with which item. This does not matter for the dissimilar condition (right), because the target is almost certainly the one with the 3° observation in this example. This cue is not available in the similar condition, making it inherently more difficult. Any cue that helps reduce that uncertainty could make that difficult condition easier.

These demonstrations suggest that an HD pooling model may at least partially explain grouping effects. In addition, some grouping effects may arise in part from noncrowding mechanisms. Many crowding experiments investigating grouping effects have a potential confound; because crowding leads to location ambiguity, we need to worry about cuing effects. For example, observers asked whether the target line tilts up or down in the dissimilar-orientation condition in Figure 7 may make use of a 100% valid cue that the line with the oddball orientation is the target. HD pooling preserves the feature dissimilarity between target and flankers that would allow the observer to make use of this cue. This cue is not available in the similar-orientation condition. An observer's noisy internal representation of the two arrays may look something like the cartoon in Figure 14. It should be obvious that the dissimilar condition is inherently easier. The observer in that condition knows to ignore the near-vertical observations, regardless of their noisy observed locations, and respond based on the one near-horizontal observation. In the similar-orientation condition, the observer lacks this information and as a result is strongly affected by the noise in all three observations. Another way to conceptualize this asymmetry is that an ideal observer also predicts that the dissimilar condition will be easier. Grouping effects in crowding may at least in part arise due to generic cuing—that is, decision-making effects—and not due to mechanisms specific to crowding or peripheral vision. In this example, crowding mainly plays a role in introducing location uncertainty, which in turn makes the oddball cue useful.

**Figure 15:**
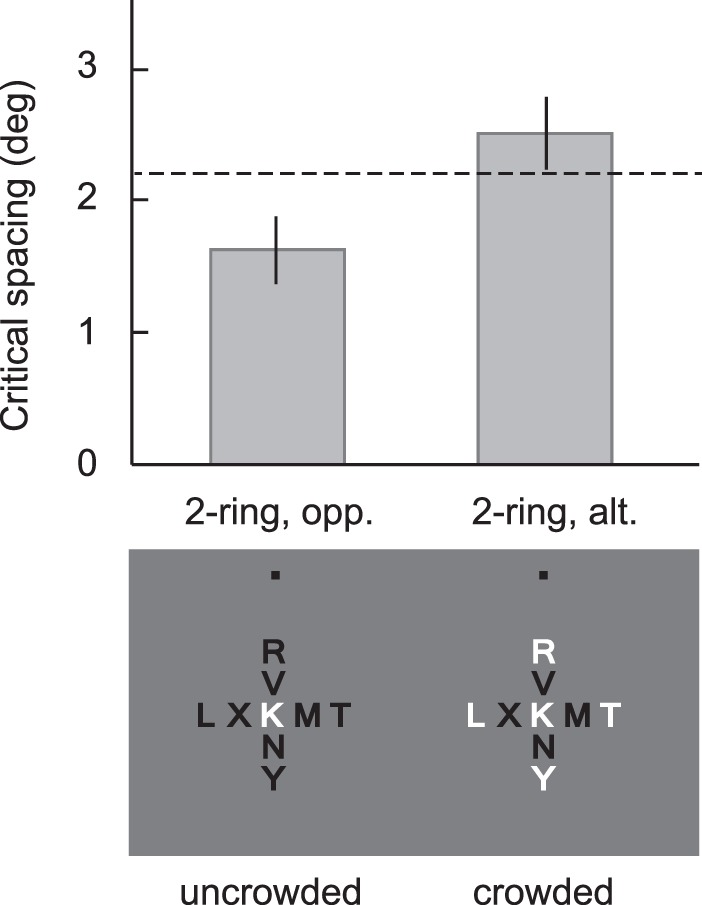
When an odd sign of contrast no longer cues the target (right), performance drops, as indicated by an increase in critical spacing. Adapted from “Crowding by a repeating pattern,” by S. Rosen and D. G. Pelli, 2015, Journal of Vision, 15(6):10, p. 4. Copyright 2015 by S. Rosen and D. G. Pelli.

Along these lines, Rosen and Pelli (2015) first replicated a sign-of-contrast similarity effect (Figure 15, left). The sign of contrast of the target was random on each trial, so the observer did not know to report, say, the white letter but did know to report the letter with the unique sign of contrast. The researchers then made the cue less useful by introducing additional rings of letters with the same sign of contrast as the target. Performance suffered.

**Figure 16:**
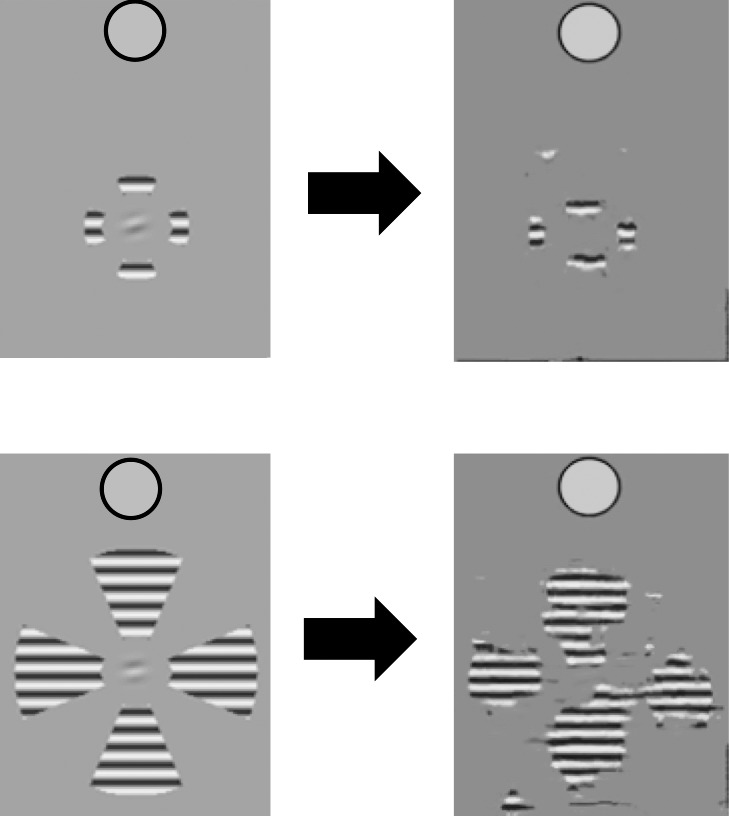
Orientation thresholds for discriminating the orientation of the target are lower when the flankers are large wedges compared to small wedges, for a fixed inner diameter of the flankers. Stimuli from Levi and Carney (2009) are to the left of the arrows. Mongrels for these two conditions (to the right of the arrows) preserve the distinction between large and small flankers but obscure the target. This suggests both a potential cuing effect and room for improvement in the model. Original stimuli reprinted with permission from “Crowding in peripheral vision: Why bigger is better,” by D. M. Levi and T. Carney, 2009, Current Biology, 19, p. 1989. Copyright 2009 by Elsevier.

In related work, Levi and Carney (2009) varied flanker size, number, spacing, distance to closest point of the flanker, and so on. They asked observers to identify the orientation of a Gabor target flanked by a variety of windowed gratings. Figure 16 shows example stimuli from a manipulation that varies the outer diameter of the window while keeping the inner diameter fixed. Over a range of conditions, the researchers found that crowding strength depended upon the spacing between the target and flanker centroids, not on the amount of blank space between them. If crowding mechanisms are sensitive to object centroids, this implies that they operate on objects, or at least after object segmentation has occurred. This would challenge typical pooling accounts, as it suggests that the critical mechanisms operate later than the presumed feature-integration stage.

However, like the similarity effects already discussed, Levi and Carney's (2009) experimental conditions may be subject to cuing effects. In their critical experiment, they varied the size of the flankers while keeping the inner diameter of the flankers fixed. Performance improved as the flankers got larger. They attributed this result to the improved encoding of the target Gabor due to the increase in the center-to-center distance between the flanker and the target. However, larger flankers also look considerably less like the target (Figure 16, left). If peripheral vision preserves this information, then observers might use it to counteract location uncertainty—they should report of the orientation of the small item—leading to improved performance. The mongrels in Figure 16 show that an HD pooling model can capture the difference in flanker size between the two conditions, suggesting that peripheral vision may preserve the information necessary to provide a cue to the target. Levi and Carney's complex pattern of results may arise from a mix of classic crowding (poorer encoding when target and flankers lie closer together) and cuing effects. Determining whether cuing effects influence these results requires experimental verification. It would be premature to claim an object-based crowding mechanism without ruling out this confound.

The mongrels in Figure 16, however, certainly suggest room for improvement in our candidate HD pooling model (or at least in the optimization process that generates the mongrel images). The representation appears to lose the information necessary to report the target orientation. The model may, for instance, need to better mimic contrast sensitivity mechanisms so as to better represent the low-contrast target.

**Figure 17:**
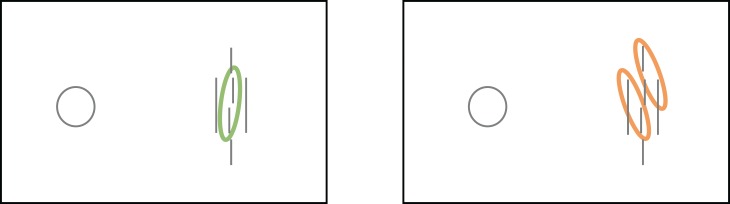
Vernier acuity tasks require the correct placement of feature detectors. Placing the feature detector like in the left panel gives observers the right answer, while placing detectors like in the right panel leads to wrong answers.

Vernier acuity tasks, from Herzog and colleagues (Malania, Herzog, & Westheimer, 2007; Manassi, Sayim, & Herzog, 2012; Sayim, Westheimer, & Herzog, 2010), also have the flavor of cuing effects. Vernier acuity requires a decision based on precisely placed feature detectors. In the example in Figure 17, the green detector gives the right answer, whereas the somewhat misplaced orange detectors would both give the wrong answer. The Manassi et al. (2012) experiments provided an explicit location cue (the lines above and below the vernier), but peripheral pooling can disrupt the location of that cue.

**Figure 18:**
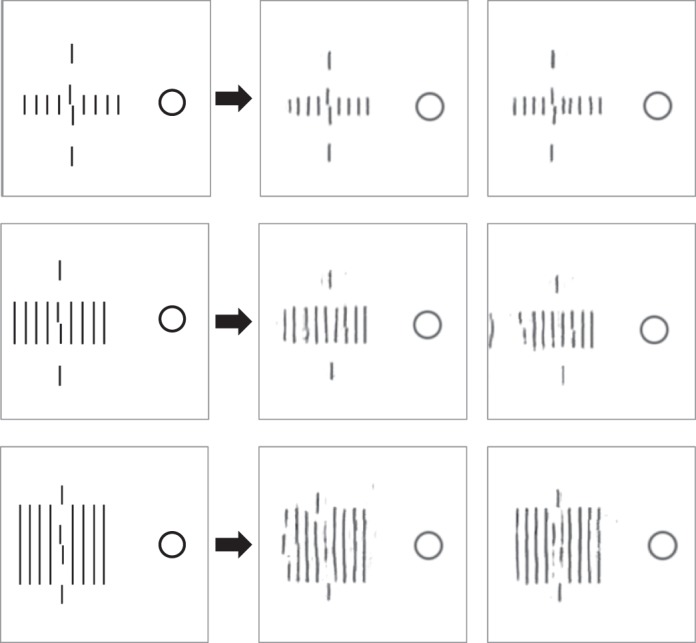
Three conditions from Manassi, Sayim, & Herzog recreated based on descriptions from the methods section (2012; stimuli to the left of the arrows, mongrels to the right). The observer's task is to discriminate the direction of offset of the vernier. The bottom condition is easy and the middle condition difficult, and in both performance is independent of the number of flankers. Increasing the number of flankers in the top condition improves performance, perhaps because more flankers leads them to group into an extended object clearly distinct from the target vernier pair. The mongrels show that sufficient information survives pooling both to distinguish the direction of vernier offset and to provide a cue distinguishing flankers from vernier in the easier conditions.

Let us further examine the three conditions from Manassi et al. (2012) as an example (Figure 18). In the bottom condition, the vernier pair looks quite different from the long flankers, and observers can use this difference to reduce uncertainty; they know to respond to the apparent tilt of the short item. This condition should always be easy, regardless of the number of flankers, as was found by Manassi et al. In the middle condition, the vernier pair looks like the flankers, removing a length cue. That condition should always be hard, regardless of the number of flankers—which it is. In the condition on the top, more flankers may form a better group, which in turn may provide a better cue to help localize the vernier. This would suggest better performance with more flankers, as was found by Manassi et al.

In many ways this explanation of the Manassi et al. (2012) effects parallels the researchers' own interpretation. They demonstrated that grouping strength plays a large role in task performance, and suggest either that crowding mechanisms might operate later in visual processing than grouping mechanisms or that information from grouping mechanisms feeds back to crowding mechanisms, dynamically adapting those mechanisms. We agree that grouping plays a big role, but attribute that role to providing a cue rather than to dynamically adapting the mechanisms of crowding. In fact, several recent studies have modeled the crowded-vernier task results either almost solely with a decision mechanism (S. Zhang, Song, & Yu, 2015) or with grouping processes alone and no special peripheral processing (Francis, Manassi, & Herzog, 2016). It is notable that the original work of Malania et al. (2007) demonstrated similar effects in both fovea and periphery; the observed effects may be only minimally due to crowding per se.

Figure 18 shows a pair of typical mongrels for each condition. Here the question is not whether the target is more poorly represented in the difficult conditions—though that may be true in some cases—but rather whether the representation preserves enough information about both the vernier offset and the grouping structure to support the use of grouping as a cue. Both seem to be true. However, we note that more recent work has found that for some of the vernier stimuli, TTM seems unable to predict the grouping effects; more flankers lead to worse representation of the target (Doerig, 2019).

**Figure 19:**
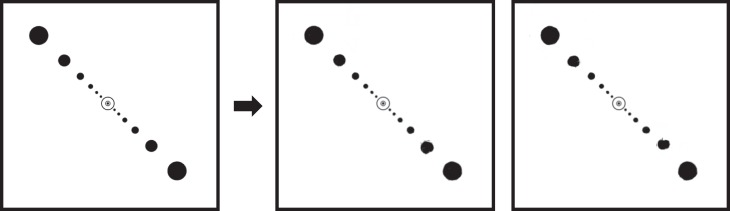
Radial tracking task from Intriligator and Cavanagh (2001). Original image, based on their stimuli, on left; on right, two typical mongrels. White circle indicates 1° at fixation. Note that pooling preserves the progressive increase in disk size fairly well.

Having discussed the potential for cuing confounds, we should revisit the work of Intriligator and Cavanagh (2001). Recall that they varied the spacing of a number of disks until observers reached threshold performance in tracking the verbally cued item. When the disks were arrayed in an isoeccentric circle about fixation, the critical spacing closely matched that of crowding. However, when the researchers instead asked observers to track the indicated disk among others arranged radially (Figure 19), they found a critical spacing that was smaller than in a traditional crowding task. Importantly, the disks in the radial task varied in size with eccentricity—an attempt to control for cortical magnification. This variation in size, however, likely provided an additional cue that observers could use to keep track of the attended item. If so, this would explain the smaller-than-expected critical spacing in the radial tracking task.

In summary, it appears plausible that a static HD pooling mechanism could explain some of the grouping effects previously attributed to a flexible pooling mechanism. Some grouping effects, in addition, may arise from cuing confounds rather than from crowding per se. An HD pooling mechanism loses some information and maintains other information. Processing then continues, acting on the available information. This includes later grouping processes as well as a decision stage that makes use of both perceptual organization and top-down knowledge to disambiguate the target and perform the task. More work needs to be done first to control for potential cuing confounds and then to quantitatively test static HD pooling mechanisms on a wide range of phenomena before ruling out such mechanisms in favor of more complicated flexible pooling.

## Challenge 4: High-level information survives crowding

In standard pooling models of crowding, the pooling supposedly occurs over fairly low-level features. For example, in our candidate HD pooling model, many of the statistics computed involve pooling at something like a junction-processing stage. Empirical studies have nonetheless found evidence that higher level information can survive crowding. Such results might seem at first glance to preclude the possibility that crowding derives from a low-level pooling mechanism.

**Figure 20:**
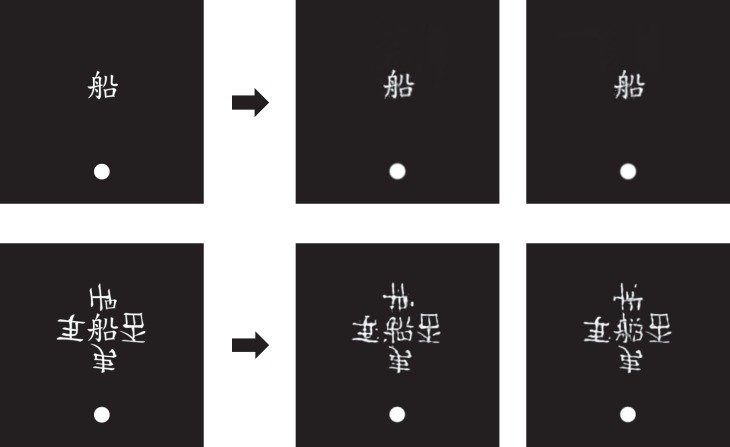
Stimuli from Yeh, He, and Cavanagh (2012) to the left side of the arrow, and mongrels for those original stimuli. High-definition pooling preserves single characters very well. Encoding of crowded characters is ambiguous but preserves some information—for example, some radicals. Thanks to Su-Ling Yeh for providing example stimuli that were not previously published.

For instance, Yeh et al. (2012) have found that Chinese characters that cannot be identified under conditions of crowding (Figure 20) nonetheless can prime a word/nonword lexical decision task when the word has a meaning related to that of the crowded character. The unidentified character can speed responses by about 50 ms compared to trials on which the characters are not semantically related. Surprisingly, they found no significant difference in the magnitude of the priming effect for a crowded versus an uncrowded character, suggesting that significant semantic information about the crowded word survives despite the inability to identify it.

It is true that a *low-dimensional* pooling model would degrade the available information so as to render higher level processing nearly impossible. Consider for the sake of argument a model that encodes the visual input using only the mean of a single feature. The loss of information would be *profound*. Such a model could clearly predict difficulty identifying a crowded target. But it would also predict poor performance at nearly all peripheral tasks. How would an observer ever identify anything, even an isolated single letter? However, an HD pooling model behaves fundamentally differently. High-dimensional pooling preserves far more information about the stimulus and can support many tasks. The mongrels shown in this article demonstrate that sufficient information survives for higher level processes to make rough estimates of the number (Figure 3) and size (Figure 16) of objects presented, detect feature pop-out (Figures 7 and Figure 8), construct perceptual groups (Figure 18), and form coarse representations of shapes (Figure 9) and letters (Figure 13). Previous work has shown that the available information suffices for some symbol-identification tasks, scene perception, and visual search (Balas et al., 2009; Ehinger & Rosenholtz, 2016; Keshvari & Rosenholtz, 2016; Rosenholtz, Huang, Raj, et al., 2012). Losses from an HD pooling model do not rule out later processing.

Can a pooling model preserve enough information about the target to prime a lexical decision task while still leading to poor identification performance? While this certainly seems a challenge, it is not out of the question. Identifying a Chinese character requires accessing sufficient information to distinguish it from a large number of alternatives. On the other hand, we do not know how much information is required to obtain a priming effect. Plausibly, portions of a crowded character might survive pooling—not enough to produce correct identification, but sufficient to provide some information about the meaning. Figure 20 shows that mongrels preserve a fair number of details about the crowded character, for example the radical “
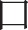
”. There also may be enough information to identify the radical “
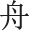
”. Radicals are characters that either carry meaning when they appear by themselves or are common subparts in a family of characters that may share similar meaning. For example, the target character in Figure 20 means “boat,” and its radical, “
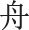
”, is commonly associated with other characters related to boats as well. Seeing this radical alone might be able to elicit representations associated with boats, thus allowing for a priming effect. Yeh et al. (2012) intentionally picked for their lexical decision task characters for which the radical did not match the meaning of the word, thus avoiding the most obvious confound. Nonetheless, the perception of such radicals may elicit some semantic processing. Observers were approximately 25% correct at the crowded-character recognition task, whereas chance performance would be far lower. Though the researchers examined priming only on trials on which object recognition failed, we should not assume that observers had no information about the target on those trials. Rather, 25% correct performance may imply that observers could narrow the answer to four possibilities. Perhaps all four choices activated some semantic information, leading to priming while prohibiting correct performance at the harder character-identification task. While it seems hard to imagine that the magnitude of priming would be as great as with correct identification of an uncrowded target, it would nonetheless be interesting to examine these conditions to ask what information about Chinese characters survives crowding, and whether TTM preserves that information.

More generally, given our presumption that processing continues after pooling, we expect task to matter. In an earlier section, we discussed this in regard to set perception. Lacking the information to identify a target in a crowded array does not mean one lacks all information about that target, as if one had failed to select the target and therefore failed to process it. Low-level crowding may permit some higher level information to “get through the bottleneck of crowding” (Fischer & Whitney, 2011, p. 1389). One may, for example, have sufficient information about the target for it to influence perception of the mean. Nor does ability to perform a task imply that the observer has full information about the stimulus—that is, that no crowding has occurred. One could perform a target/nontarget face task in the periphery (Louie et al., 2007) and yet not preserve sufficient information to support a more fine-grain identification of that face among 100 possible choices. Performance can differ on two tasks because they require different information or have different inherent difficulty.

## Conclusions

Pooling models of crowding have been popular under a number of different names, from faulty integration through compulsory averaging to forced texture perception. Recent empirical results have appeared, on first examination, to challenge a pooling account of crowding. This suggestion, however, has arisen in large part from attempts to gain intuitions about an inherently high-dimensional representation by extrapolating from simple low-dimensional models. Intuitions about low-dimensional models notoriously do not generalize well to higher dimensions. One of our goals in this article is to provide better intuitions about HD pooling models than one can acquire from introspecting based on low-dimensional straw-man models, and based on those intuitions to reevaluate the seriousness of the challenges to pooling models. High-dimensional pooling preserves enough information to recognize features of individual elements, construct shape and perceptual groups, and make judgments about objects and scenes. This calls into question a number of the model challenges. Other apparent model challenges may arise at least in part from decision effects that are not specific to crowding. Further, more quantitative examination is required. Nonetheless, we suggest that reasonable doubt remains as to whether the challenges truly eliminate pooling models. It appears that pooling remains viable as an explanation of peripheral crowding.

At a higher level, two cautionary lessons emerge. First, one should be careful not to confuse a phenomenon with a mechanism. Substitution phenomena do not imply a substitution mechanism. Crowding by similar orientation, sign of contrast, parts, shapes, faces, or point-light walkers does not imply that difficulty identifying the crowded peripheral target arises from mechanisms operating at the corresponding processing stage. Second, one should avoid claiming that a model cannot explain a set of results without actually specifying and testing the model.

Examples in both this article and earlier publications clearly point to imperfections in our candidate HD pooling model, TTM. The model lacks some obvious second-order statistics that would better capture contour integration, calling into question whether it can explain effects such as those observed by Livne and Sagi (2007), where crowding of a target Gabor was relieved if the flanking Gabors align and form a smooth contour. The model captures a good deal of end stopping and yet lacks explicit end-stopping features. Such features may be important for tasks such as distinguishing an array of Os from one of Os and Cs. Lastly, readers might remember that the target was altogether lost in the mongrels for stimuli from Levi and Carney (2009), suggesting at minimum a failure to handle low-contrast information for these stimuli (though whether the fault lies in the encoding itself or in the optimization procedure that generates the mongrels remains to be seen).

Eliminating pooling models as a class is difficult. Choosing what features to pool provides a powerful and flexible way of varying the information lost and maintained by a given model. Vary the number or complexity of features, or the areas over which the model pools them, and the information available can change in profound ways.

One might instead go so far as to think of an HD pooling model in terms of Occam's razor. It provides the (relatively) simple explanation for a range of phenomena, and as such serves as a useful check for whether more complex mechanisms are required and, if so, which ones. The Texture Tiling Model has been particularly successful in this regard, not only explaining a range of crowding results but calling into question more complex explanations for difficult visual search, change blindness, set perception, and easy scene perception (Rosenholtz, 2016; Rosenholtz, Huang, & Ehinger, 2012). Future work is needed to show quantitatively how much TTM does or does not account for the experimental findings associated with the model challenges. Nonetheless, the mongrels, which provide us with intuitive visualizations of what information is preserved by the model, show promise.

Explaining crowding phenomena may well prove to require more complicated mechanisms. The challenge for alternative models of crowding lies in capturing the range of phenomena already explained by pooling models. TTM has, to date, been tested on over 70 conditions. Pooling models have been sufficiently successful that competitors must demonstrate similar or better explanatory power for a wide range of empirical results. Gone are the days in which we can consider a new model to be viable when it explains the results of a single experiment. The model challenges reviewed in this article provide a useful test set for distinguishing between models as we move forward.

## Footnotes

1 Details about the Texture Tiling Model and relevant discussion can be found in Appendix A.

2 Models like TTM are often referred to as “texture synthesis” models, but this is a misnomer. We do not assume that the visual system “synthesizes” any textures. The texture synthesis is used only to generate members of the equivalence class of the model. It is not part of the model per se.

3 The SDT model of Morgan and Solomon (2005) corrects an important error in the original (Parkes et al., 2001) report. Correcting this modeling error does not appreciably change the conclusions of Parkes et al.

## Appendix A: The Texture Tiling Model

In the examples in this article, we use the Texture Tiling Model (TTM) as our candidate high-definition pooling model. This appendix gives a more detailed explanation of the model, along with a discussion of various decisions made in creating the model. In particular, it describes the statistics measured and the pooling regions used, and gives a brief description of the algorithm by which we generate visualizations of the information lost and preserved in peripheral vision. We have provided the MATLAB code at https://dspace.mit.edu/handle/1721.1/121152.

This article utilizes what we refer to as the full-field version of TTM. While some of our earlier articles (Balas et al., 2009; Rosenholtz, Huang, & Ehinger, 2012; Keshvari & Rosenholtz, 2016) have made predictions based on a single-pooling-region version—equivalent, to a first approximation, to Portilla and Simoncelli's (2000) texture analysis/synthesis algorithm, modified to work robustly on psychophysical displays with large blank regions—the present model utilizes information from multiple pooling regions across the visual field. Within each pooling region, TTM runs this modified Portilla and Simoncelli algorithm with the following parameters: number of scales = 4; number of orientations = 4; and, for these examples, Na = 7. The parameter Na (also referred to as M by Portilla and Simoncelli) specifies the number of central samples of the autocorrelation used as constraints. In the past we have also used Na = 9, which gives similar results for most stimuli tested. This algorithm computes the following summary statistics: marginal statistics of luminance and color; autocorrelation; correlations of responses of V1-like cells across location, orientation, and scale; and phase correlation across scales. Presuming that the visual system computes local summary statistics as hypothesized by pooling models, further investigation will be required to pinpoint exactly what statistics are involved. Though previous work has suggested that the aforementioned statistics provide a good initial guess, we would be surprised if this initial set of statistics proved to be correct. The summary statistics might involve more biologically plausible computations, or might derive from features learned for ecological tasks such as object recognition. Additional statistics might be needed to better capture contour-integration behavior, or to more explicitly compute end stopping. In addition, one would of course expect to include some statistics based on motion and on binocular disparity.

Given a fixation point, TTM tiles the image with overlapping pooling regions. For the examples in this article, we have used square pooling regions. Though we have previously implemented elongated elliptical pooling regions, the square regions allowed for faster processing and led to fewer synthesis artifacts in our hands, while otherwise not greatly differing in results from elliptical pooling regions. Square receptive fields are of course completely biological implausible. They also appear in opposition to behavioral work, which finds roughly twice the critical spacing in the radial direction as in the tangential (Toet & Levi, 1992), though such differences in critical spacing could also derive from the pattern of overlap of the pooling regions (Rosenholtz, 2016). Further work is required to determine the number, location, size, and degree of overlap of these regions necessary to best predict human behavior. The pooling regions used here have width equal to 0.5 times the eccentricity at their center. They have a radial overlap of 45%; for a pair of pooling regions arranged in a radial direction, this specifies what percentage of the width of the inner pooling region overlaps with the outer pooling region. Tangential overlap is determined by the size of the pooling regions plus the number of pooling regions in the tangential direction. The examples here use 36 pooling regions tangentially—that is, with their centers every 10° around a circle centered at fixation.

The algorithm performs two preprocessing steps. First, if one simply lays down pooling regions across the entire image, some pooling regions will land partially outside the original image, necessitating image padding. Second, prior to measurement of statistics, TTM blurs the input image to approximately mimic the loss of acuity with eccentricity.

Synthesis is initiated by assuming that a foveal region (which we conceptualize as a small 1°–2° circle about fixation) is reconstructed perfectly. For the purposes of this article, we used a default size for this fovea of 32 pixels in radius. One might loosely think of this as the number of pixels per degree. Then, moving in an outward sweep, each subsequent pooling region is synthesized using the previous partial-synthesis result as the seed for the texture-synthesis process (plus, in the first iteration, noise in regions not yet synthesized). When replacing each synthesized patch, we blend it with overlapping regions using a Gaussian distance-weighted average. A second sweep reconstructs the pooling regions from the outer rings inward, ending by reapplying the fovea. This process iterates a number of times over the entire image. We use a coarse-to-fine strategy, starting with the coarsest scale and adding one scale at a time, to speed convergence. At each stage of this coarse-to-fine procedure we run 10 iterations of synthesis of the entire image. Within each such iteration, we run three iterations of each pooling region.

Little previous work speaks to what color statistics the model should compute. It seems likely that the visual system computes summary statistics in several color channels, and perhaps also computes some sort of correlations between those channels. More research is required. Here we first use independent component analysis (Bell & Sejnowski, 1995) to split the image into three color bands. This is somewhat unrealistic in that it suggests that the computed statistics change with the contents of the stimulus, but in practice it works well. We measure statistics in each of these bands independently. Within each local pooling region we also measure the covariance between the three color bands, and apply that constraint after synthesizing the three channels of each local pooling region.

Additional details are documented with the code. This includes details involved in the functioning of the code and recommendations for running the algorithm, including choices of the number of pixels per degree and other adjustments to the parameters of the algorithm.
